# Supplementary material for: Complementary Effects of Carbamylated and Citrullinated LL37 in Autoimmunity and Inflammation in Systemic Lupus Erythematosus
Source: Int J Mol Sci. 2021 Feb 6;22(4):1650. doi: 10.3390/ijms22041650 (PMC7915858; doi:10.3390/ijms22041650)
Supplement: Supplementary file 1 [file ijms-22-01650-s001.pdf]

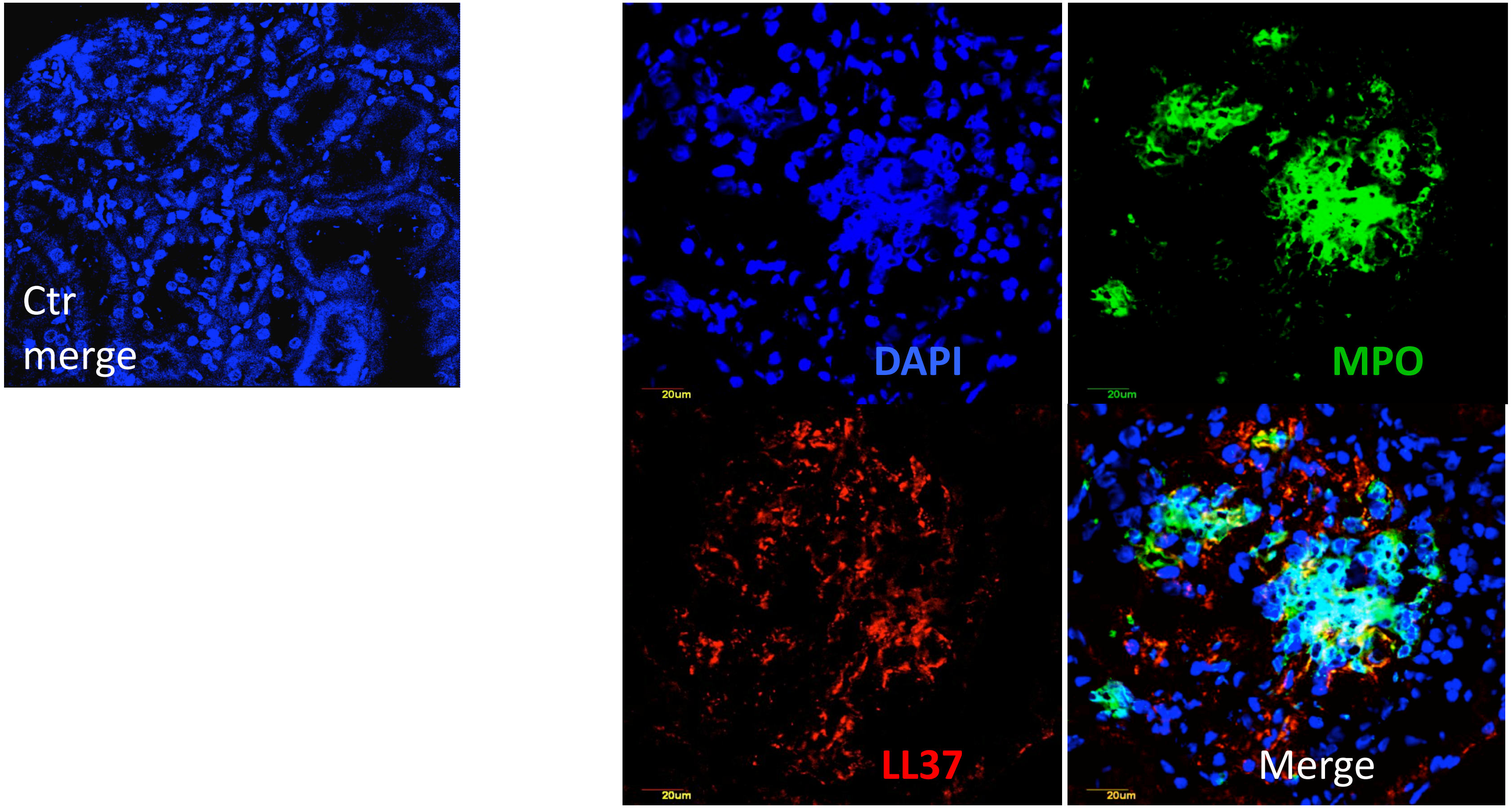

Figure S1. MPO and LL37 are present in SLE-affected kidney. Confocal microscopy images of staining of SLE kidney biopsies for presence of LL37 and MPO (magnification 40x). Images are representative of staining of sevem renal biopsies, derived from 7 different patients with lupus nephritis (class IV).

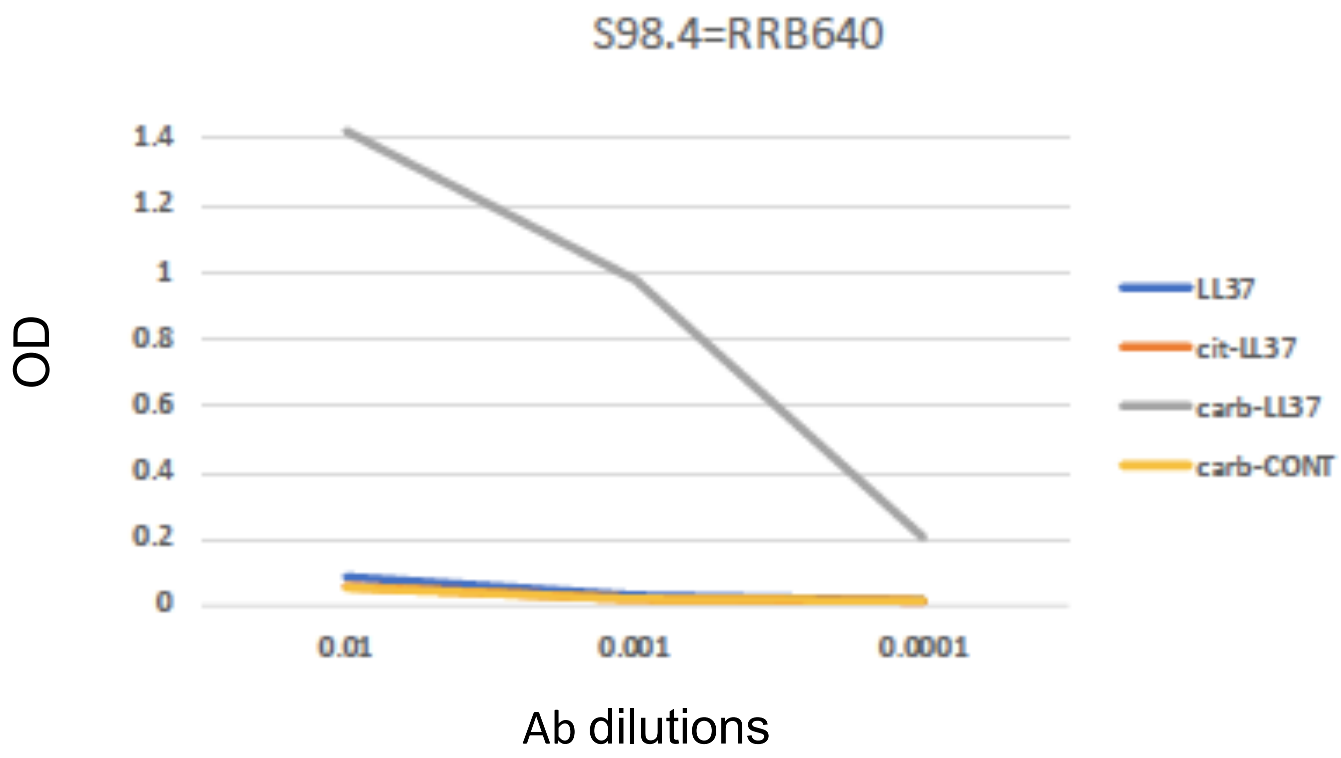

Figure S2. Mab RRB640 is specific for carb-LL37 and does not not cross-reacts to cit-LL37, native LL37 nor to a control fully carbamylated LL37 reverse peptide (carb-CONT). Dose response reactivity (expressed as Optical Density, OD) of Mab RRB640 to carb-LL37 and control peptides. Results are representative of three different experiments. Initial concentration of the Mab was 80 μg/ml.

Table S1.
Demographical/clinical characteristics of the studied SLE cohort and control individuals

Patients	SLE (50)	HD (30)
Age, mean (range) years:	43 (30–70)	50 (27–57)
Sex (M/F):	2/48	9/21
Disease duration with range:		
Months	48 (15–120)	N/A
SLEDAI	8.5 (0-31)	N/A
Inactive SLE (SLEDAI<4)	28%	N/A
Active SLE (SLEDAI>4)	72%	N/A
Severe SLE (SLEDAI≥12)	30%	N/A
Renal involvement	12%	N/A
Rush	68%	N/A
CNS involvement	0%	N/A
Arthritis	14%	N/A
ANA	80%	N/A
Anti-dsDNA	58%	N/A
Prednisolone use	50%	N/A

SLE, Systemic lupus erythematosus; HD, healthy donors; SLEDAI, SLE Disease Activity Index; ANA, Anti-nuclear antibodies; Anti-dsDNA, anti-double strand DNA antibodies; CNS, central nervous system; N/A, not applicable

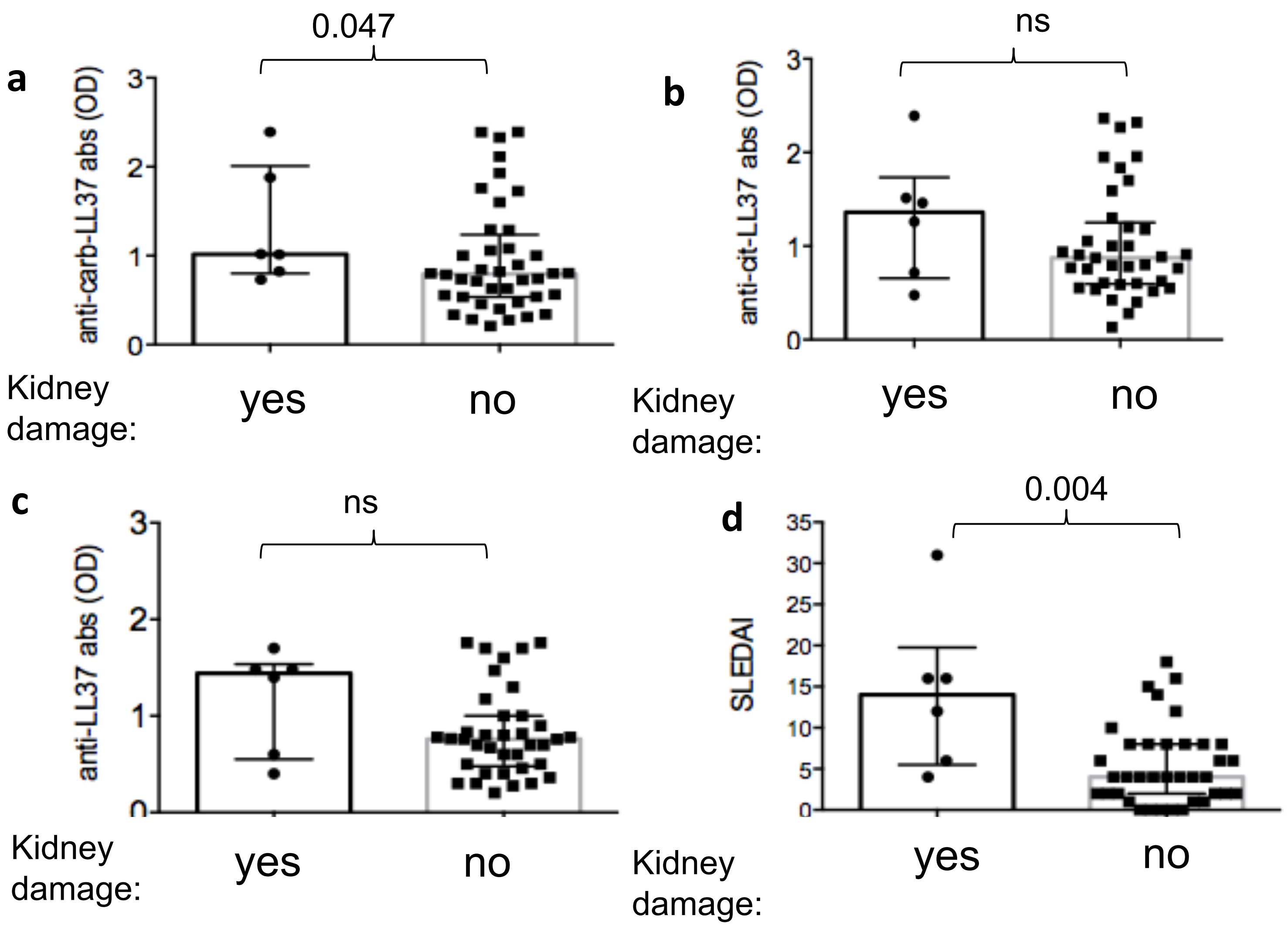

Figure S3. SLE patients with kidney damage tend to have have higher anti-carb-LL37 antibodies that SLE without kidney damage. SLE patients were sorted in the those with renal damage (N=6) and those without renal damage (N=44) and magnitude of anti-carb- (a), anti-cit- (b) and anti-native-LL37-antibodies was tested by ELISA. (d) Difference between the SLEDAI values in SLE patients with renal damage, as compared to patients with no renal damage. The horizontal bars represent the medians with interquartile range (vertical bars), P values calculated by Mann-Whitney's test.

BINDING TO DR1B1*1501

peptides	Experiment 1				Experiment 2			Average K_D (nM)
	HLA-	Bmax	K_D (nM)	R^2	Bmax	K_D (nM)	R^2	
Pos contr	DRB1*15:01	32700	10	0,996	33300	10	0,996	**10**
LL37	DRB1*15:01	36000	590	0,982	39300	490	0,972	**540**
cit-LL37	DRB1*15:01	6300	250	0,990	23300	430	0,993	**340**
carb-LL37	DRB1*15:01	12800	370	1,00	18200	330	0,991	**350**

BINDING TO DRB1*0101

peptides	Experiment 1				Experiment 2			Average K_D (nM)
	HLA-	Bmax	K_D (nM)	R^2	Bmax	K_D (nM)	R^2	
Pos contr	DRB1*01:01	23600	80	0,998	29700	95	0,997	**87**
LL37	DRB1*01:01	7900	80	0,988	8000	55	0,989	**68**
cit-LL37	DRB1*01:01	28900	610	0,998	28700	750	0,999	**680**
carb-LL37	DRB1*01:01	30000	530	0,996	34500	810	0,998	**670**

Table S2. Carb-LL37 binds HLA-DR alleles similarly to cit-LL37. Binding of the peptides, included LL37 to the indicated HLA-DR alleles was evaluated by HLA-DR folding assay, and detected by luminescent oxygen channeling immuno-assay, as in Methods and ref. 6. a average K_D low indicate better binding affinity. Results of two experiments and their mean average are reported for each peptide and each HLA-DR allele.

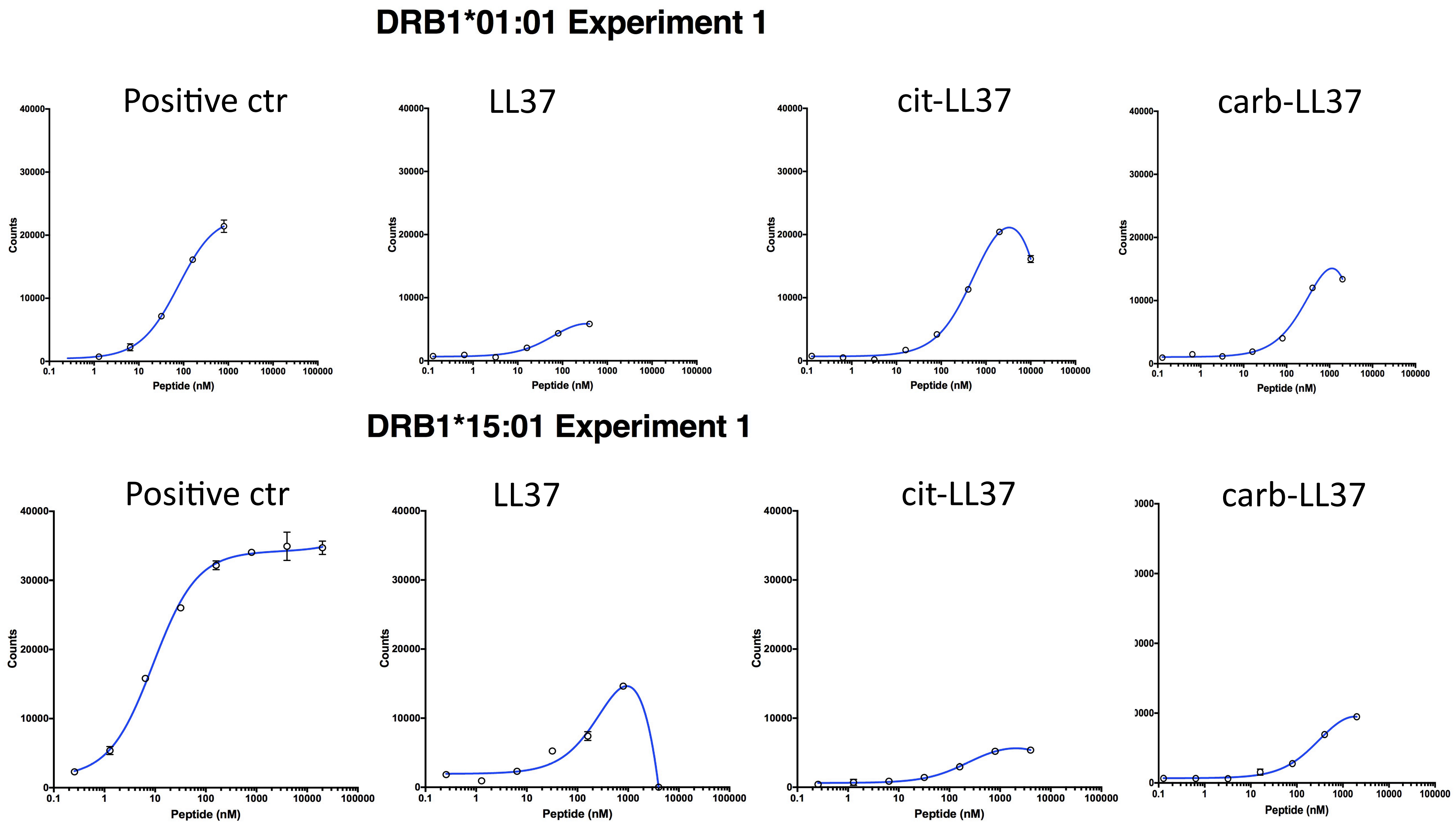

Figure S4. Carb-LL37 binds HLA-DR alleles similarly to cit-LL37. Binding of the peptides: Representative experiments showing the binding curves of each peptides.

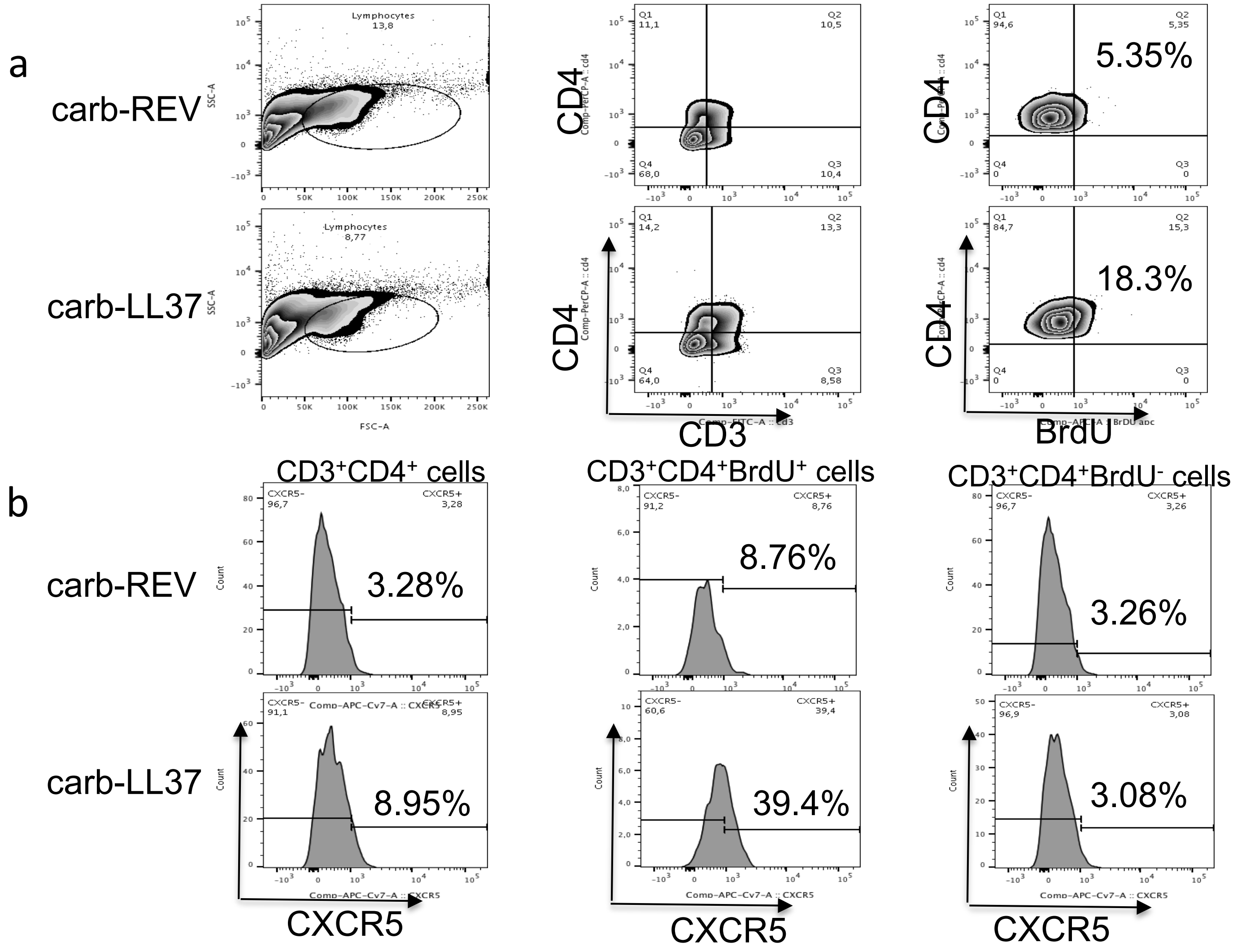

Figure S5. Gating strategy for assessing T-cell proliferation to carb-LL37 and control antigen. (a) Gating strategy to assess proliferation of CD4 T-cells (CD3+CD4+ cells) by BrdU incorporation, (BrdU), as percent of Brdu staining in CD3+CD4+ cells, to control antigen (carb-REV) or carb-LL37. Results are reported as flow cytometry zebra plots and are representative of different stainings. **(b)** CXCR5 expression on CD4 T-cells that are BrdU+,or BrdU-,(middle panels and right panels, respectively), as compared to CXCR5 expression by total CD4 T-cells (left panels), in response to either control antigen (carb-REV) or to carb-LL37.

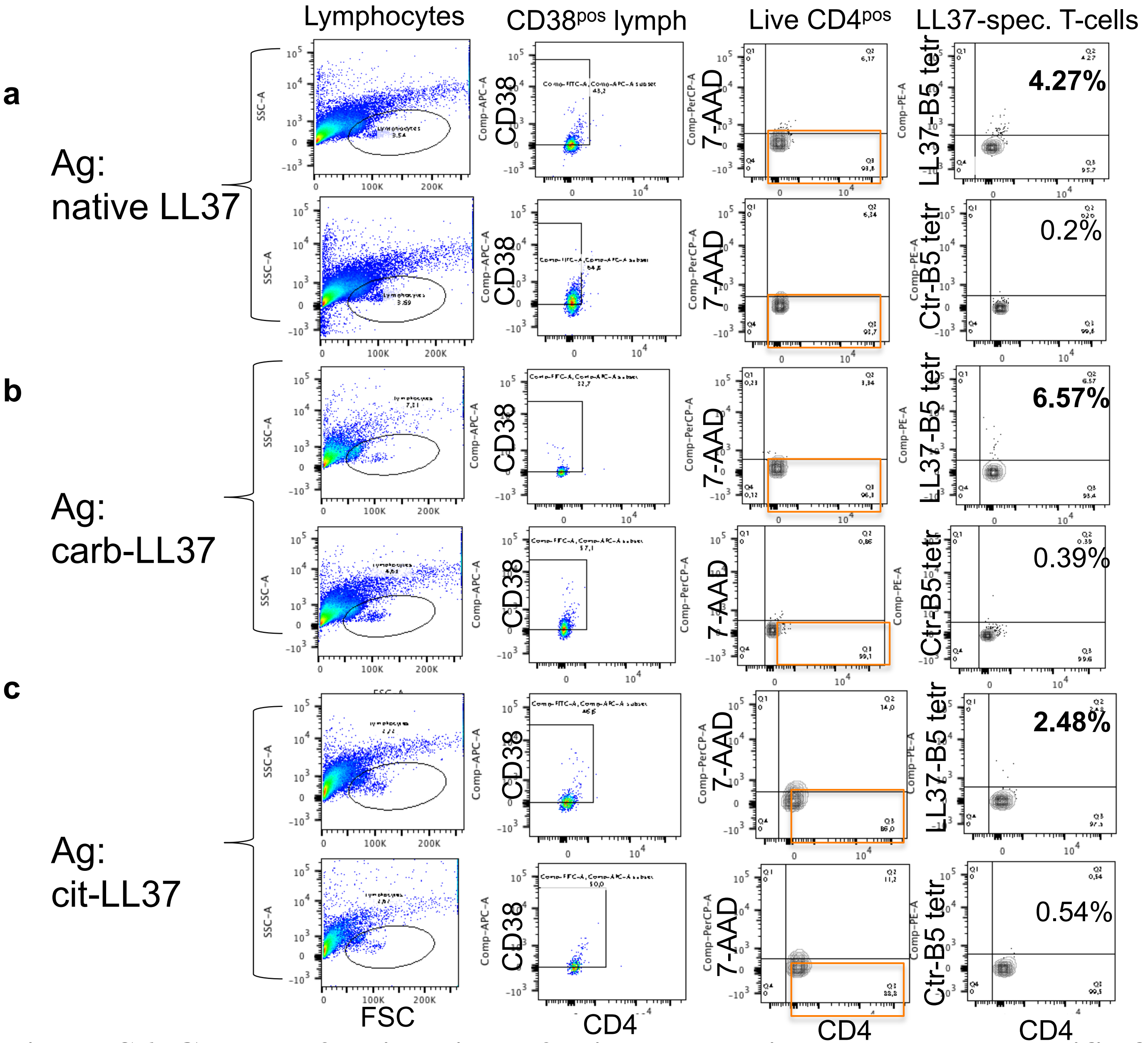

Figure S6. Carb-LL37, like cit-LL37, is able to stimulate T-cells specific for the native LL37. T-cells were stimulated for 6 days with the indicated antigens and stained with peptide-MHC-tetramers loaded with a native LL37 epitope (upper panels in **a,b,c**) or with control tetramers (lower panels in **a,b,c,**). Cells were gated on $CD38^{hi}$ CD4 T-cells (activated T-cells) and dead cells were excluded by 7-AAD-staining .Results are reported as flow cytometry dot plots.

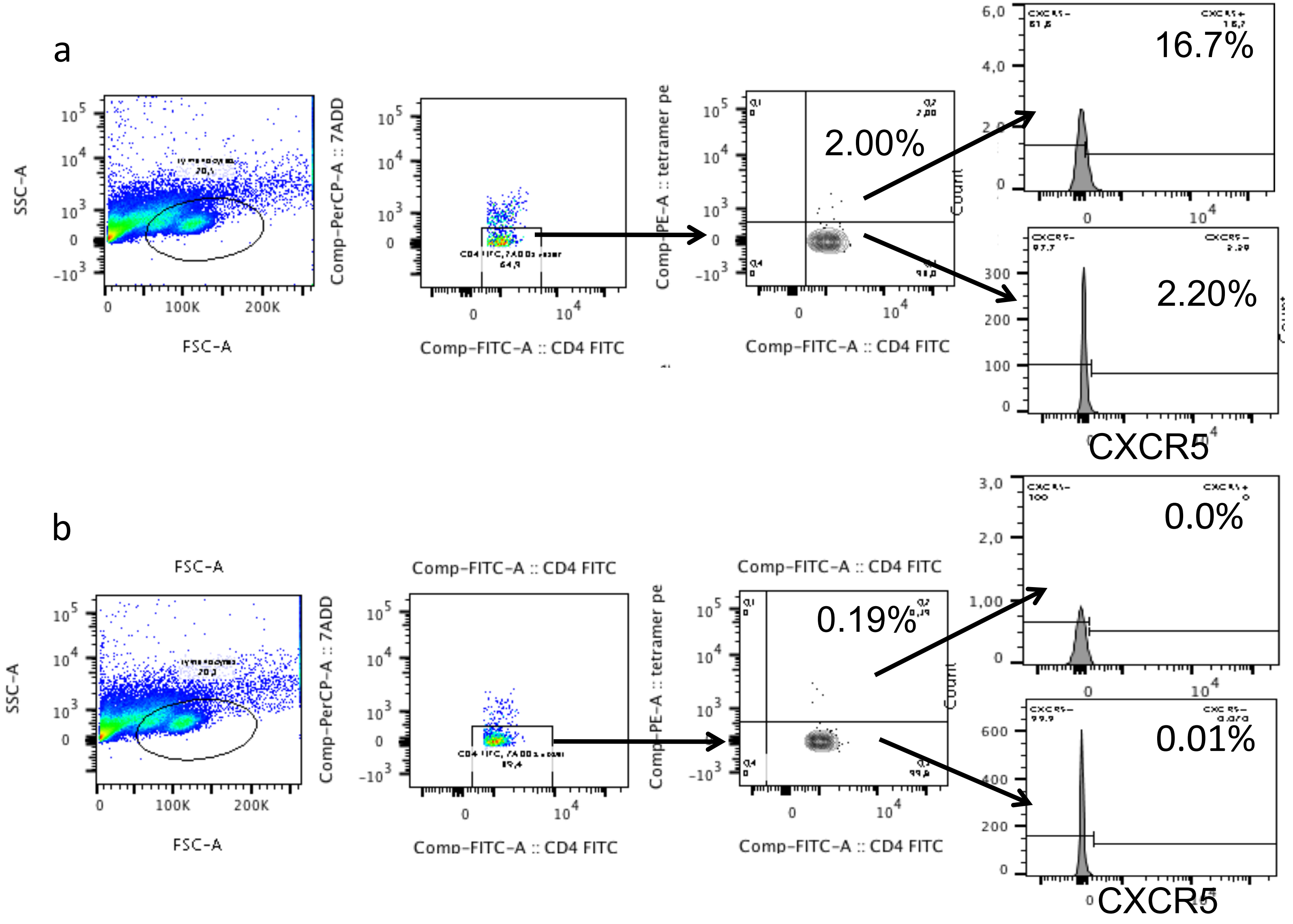

Figure S7. Carb-LL37 is able to stimulate T-cells specific for the native LL37, which express CXCR5. SLE PBMC were stimulated for 7 days with carb-LL37 and stained with peptide-MHC-tetramers loaded with the native LL37 epitope P1 **(a)** or with control peptide-MHC-tetramer **(b)**. Results are reported as flow cytometry dot plots. Gating stratetegy includes exclusion of CD4 T cells positive for 7-AAD, to eliminated dead cells. Tetramer positive and negative CD4 T-cells were assessed for expression of CXCR5, as depicted.

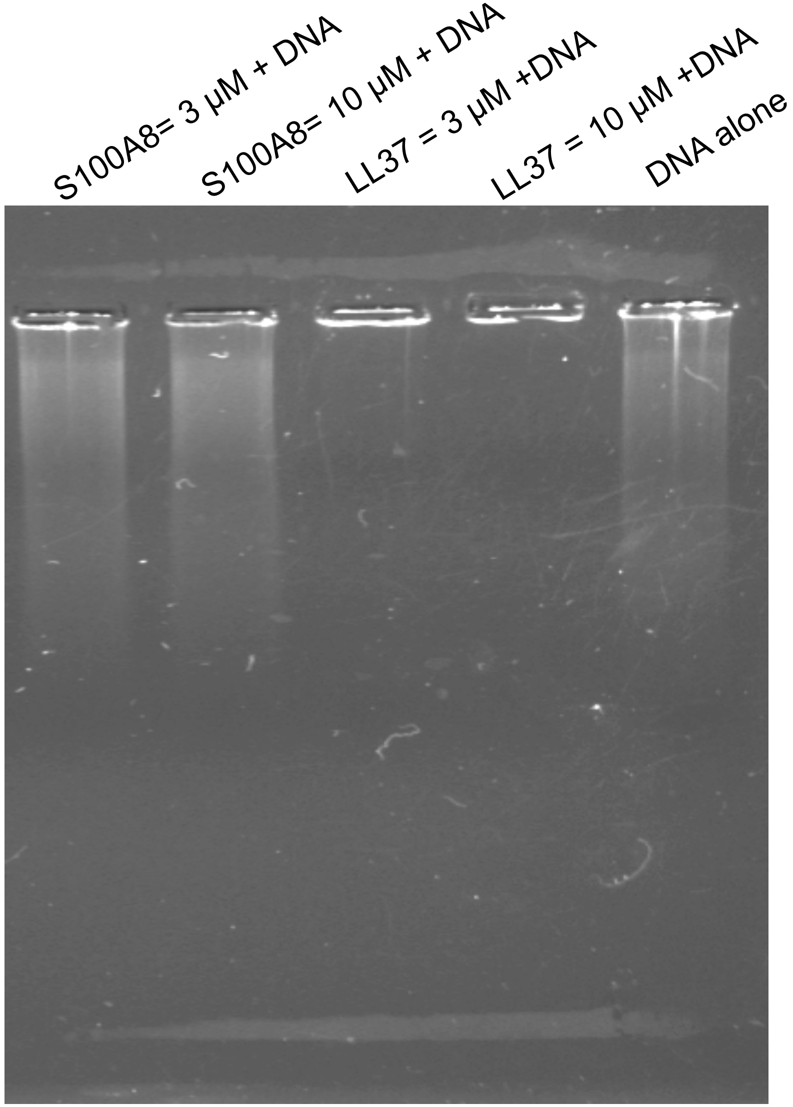

Figure S8. LL37 binds to DNA and blocks its migration on an agarose gel. DNA was mixed with LL37 or with S100A8 (negative control (ref 34), or left alone, and run on a agarose gel. LL37 binds DNA and almost completely blocks its migration on the gel. One representative experiment of four performed.

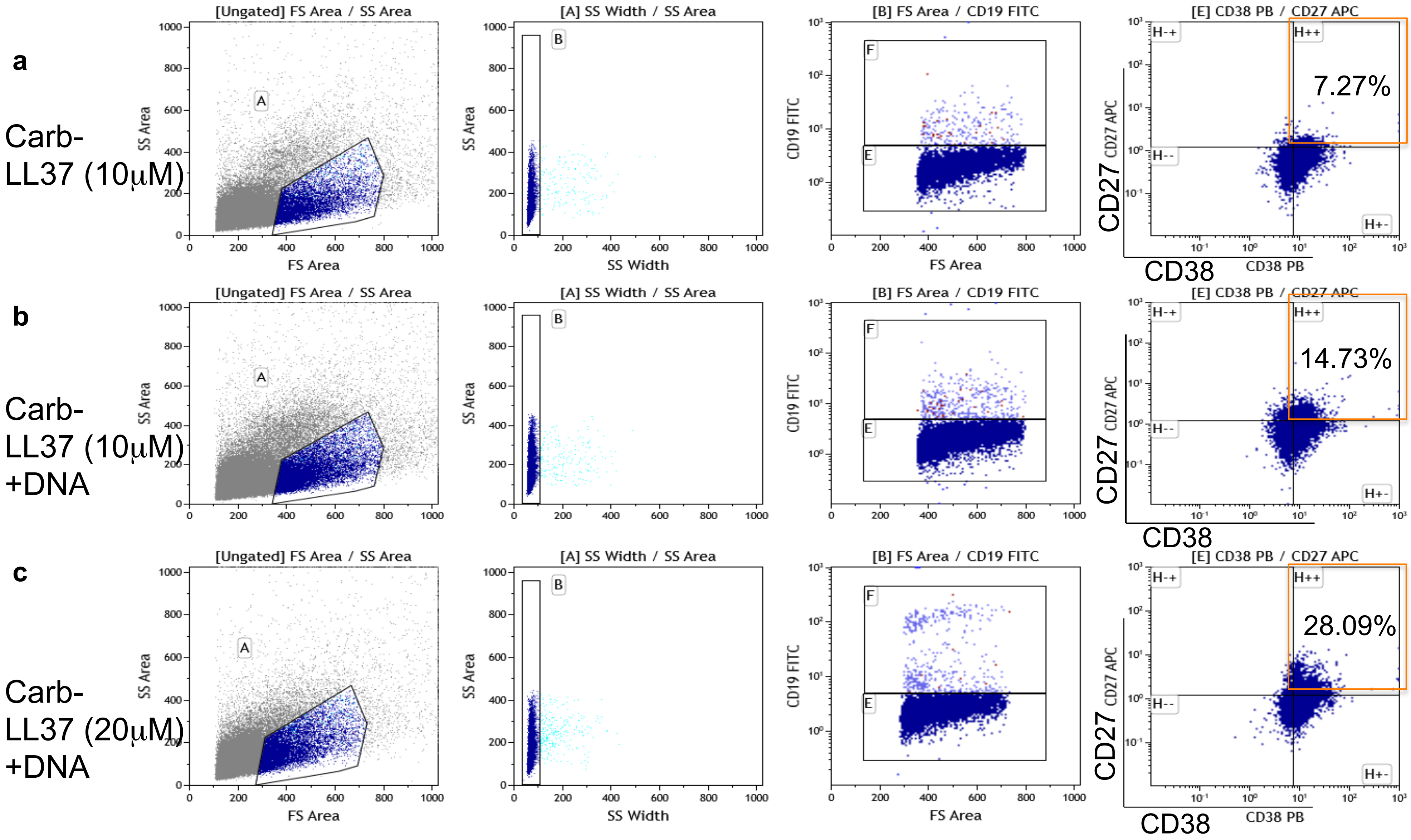

Figure S9. Carb-LL37 is able to stimulate maturation of B-cells in plasmacells in complex with DNA. Purified B-cells were cultured for 7 days with the stimuli indicated in **a,b,c** in the presence of 50UI/ml of hrIL-2. At the end of the culture, percent of $CD38^{high}CD27^{high}$ $CD19^{neg}$ plasma was evaluated by flow cytometry.
